# Evaluation of Antioxidant and Cerebroprotective Effect of *Medicago sativa* Linn. against Ischemia and Reperfusion Insult

**DOI:** 10.1093/ecam/neq019

**Published:** 2011-03-13

**Authors:** Kundan Singh Bora, Anupam Sharma

**Affiliations:** ^1^L.R. Institute of Pharmacy, Solan 173 223, India; ^2^University Institute of Pharmaceutical Sciences, Panjab University, Chandigarh, India

## Abstract

Antioxidants have been the focus of studies for developing neuroprotective agents to be used in the therapy for stroke, which is an acute and progressive neurodegenerative disorder. *Medicago sativa* (MS) has a long tradition of use as ayurvedic and homoeopathic medicine in central nervous system disorders. The plant has been reported to have antioxidant, anti-inflammatory and antidiabetic effects. Therefore, the present study was designed to investigate the neuroprotective effect of methanol extract of MS on ischemia and reperfusion-induced cerebral injury in mice. Bilateral carotid artery occlusion (BCAO) for 15 min followed by 24-h reperfusion, resulted in significant elevation in infarct size, xanthine oxidase (XO) activity, superoxide anion (O^•−^_2_) production and thiobarbituric acid-reactive substance (TBARS) levels, and significant depletion in endogenous antioxidant [reduced glutathione (GSH), superoxide dismutase (SOD) and total tissue sulfhydryl (T-SH) groups] systems in mice brain. Further, BCAO led to impairment in short-term memory and motor coordination. Pre-treatment with MS (100 or 200 mg kg^−1^, p.o.) markedly reduced cerebral infarct size, XO, O^•−^_2_ and TBARS levels, significantly restored GSH, SOD and T-SH levels and attenuated impairment in short-term memory and motor coordination. In addition, MS directly scavenged free radicals generated against a stable radical 1,1-diphenyl-2-picrylhydrazyl and O^•−^_2_ generated in phenazine methosulphate-nicotinamide adenine dinucleotide systems, and also inhibited XD/XO conversion and resultant O^•−^_2_ production. The data from this study suggest that treatment with MS enhances the antioxidant defense against BCAO-induced global cerebral ischemia and exhibits neuroprotective activity.

## 1. Introduction

Reactive oxygen species (ROS) are involved in cerebral ischemia, particularly in ischemia and reperfusion (I/R) [1, 2]. Brain is the most susceptible organ to the damage due to oxidative stress because neurons are rich in polyunsaturated fatty acids, and levels of endogenous antioxidant enzymes [superoxide dismutase (SOD), catalase, glutathione peroxidases] and none enzymes (vitamins C and E) in neuronal tissue are low [3, 4]. Therefore, oxidative stress may contribute to neuronal cell death due to I/R. During I/R insult, a number of events that predispose the brain to the formation of ROS may occur. After reperfusion, these events can set off a cascade of other biochemical and molecular sequelae such as the xanthine dehydrogenase/xanthine oxidase (XD/XO) conversion, leading to production of ROS [5]. Free radicals are important pathophysiological mediators of cell injury in stroke [6]. Oxygen radicals, such as superoxide anion (O^•−^_2_), peroxynitrite (ONOO^−^), hydrogen peroxide (H_2_O_2_) and hydroxyl radical (OH**^•^**) are normally produced in very low amounts by activated microglia and endothelial cells as products of mitochondrial metabolism [7]. Among the ROS, O^•−^_2_ was believed to be directly toxic to neurons since it initiates a free radical-mediated chain reaction causing additional central nervous system (CNS) damage [8]. In addition, total sulfhydryl (T-SH) oxidation, mediated by free radicals released during ischemia, may also contribute to XD/XO reversible oxidative conversion. Both mechanisms were suggested to cause a conformational change in flavine adenine dinucleotide domain of the XD leading to the formation of XO [9].

Since reperfusion injury is associated with an imbalance of oxidative stress and antioxidant defense system, theoretically it would be possible to limit oxidative damage and ameliorate disease progression by supplementing antioxidants [10]. Indeed, several natural and synthetic antioxidants have shown neuroprotective effect in I/R-induced cerebral injury [11, 12]. Although standardized extract of *Ginkgo biloba* (EGb761) [13], edaravone [14] or curcumin [15] have been demonstrated to be antagonistic to brain I/R, the anti-I/R agents available are still far from sufficient. In recent years, considerable interest has been generated on a leguminous plant *Medicago sativa* (MS) (Leguminosae), which is one of the most reputed medicinal plant traditionally used to improve the memory, to cure kidney pain, cough, sore muscles [16], as rejuvenator, antidiabetic, antioxidant, anti-inflammatory, antimicrobial and in CNS disorders [17, 18]. Moreover, MS has a long tradition of use as ayurvedic and homoeopathic medicine in CNS disorders [19]. Phytochemical reports on MS indicate that the plant contains flavonoids [20], alkaloids [21, 22], phytoestrogens, coumarins, digestive enzymes, triterpenes [23], saponins [24] and phytosterols [23, 25]. Several clinical and animal studies indicate that the ingestion of MS reduces cholesterol absorption and atherosclerotic plaque formation in the arteries [26, 27]. MS is beneficial in cardiovascular complaints [23], convalescence and debility [22], diabetes [28] and also when used as a tonic after blood loss and during anemia [22]. The plant has been shown to have anti-tumor activity against certain types of leukemia cells in mice and selective toxicity in dog cancer cells grown *in vitro* [29].

As reported, MS possesses antioxidant, anti-inflammatory and antidiabetic activity. However, no work has ever been carried out to evaluate the neuroprotective effect of MS on cerebral stroke. Thus, it was considered worthwhile to investigate the effect of MS on global cerebral I/R-induced cerebral injury in mice. In addition, direct free scavenging activity of MS was also evaluated against free radicals generated against a stable radical 1,1-diphenyl-2-picryl-hydrazyl (DPPH), and O^•−^_2_ radical generated in phenazine methosulphate (PMS)-nicotinamide adenine dinucleotide (NADH) systems comparable to butylated hydroxytoluene (BHT) and butylated hydroxyanisole (BHA), to assess its antioxidant effect.

## 2. Methods

### 2.1. Animals

Swiss albino mice of either sex (20–30 g) were employed in the present study. The animals were maintained on standard environmental conditions and fed with standard rodent diet (Kissan Feeds Ltd, Mumbai, India) and tap water *ad libitum*. The experimental protocol was approved by the Institutional Animal Ethics Committee and care of the animals was carried out as per the guidelines of the Committee for the Purpose of Control and Supervision of Experiments on Animals (CPCSEA), Ministry of Environment and Forest, Government of India.

### 2.2. Reagents and Chemicals

Chloral hydrate was obtained from Reidel-deHaen, Germany. Thiobarbituric acid (TBA), triphenyltetrazolium chloride (TTC), 1,1,3,3-tetraethoxy-propane, dithiothreitol (DTT), phenylmethylsulfonyl fluoride (PMSF), xanthine, XO, nitroblue tetrazolium (NBT), PMS, 5,5′-dithiobis-(2-nitrobenzoic acid) (DTNB), DPPH, reduced form of NADH, ascorbic acid, BHA and BHT were purchased from Sigma (Sigma-Aldrich GmbH, Sternheim, Germany).

### 2.3. Preparation of MS

Aerial parts of the plant MS were procured from Himalaya Herbs Stores, Saharanpur, India. Identity of the plant material was authenticated by the National Institute of Science Communication and Information Resources (NISCAIR), New Delhi vide their letter no. NISCAIR/RHMD/Consult/2008-09/1170/202 dated 25 February 2009.

Dried aerial parts of the plant were pulverized using a mechanical grinder. Powdered material (100 g) was extracted with methanol (24 h) using the Soxhlet apparatus. Thereafter, the resulting methanol extract was reduced *in vacuo* (40°C; N_2_ stream), freeze-dried and stored at 4°C until further use in the experiment. The yield was 11.2% (w/w). Phytochemical screening [30, 31] of the extract revealed the presence of flavonoids, alkaloids, phytosterols, coumarins, polyphenols and amino acids.

### 2.4. Experimental Protocols

A total of five groups of 16 mice each were employed in the present study. To obtain maximum data from a small number of animals, half the animals from each group were subjected to estimate infarct size, XO, O^•−^_2_, thiobarbituric acid-reactive substance (TBARS), reduced glutathione (GSH), SOD and T-SH levels. The remaining half of the animals from each group was subjected to elevated plus-maze test and inclined beam walking test before global cerebral ischemia and after 24-h reperfusion. Vehicle or drugs were fed once daily for 10 consecutive days prior to the experiment and treated as follows.

- Group I: Sham group (*n* = 8). Mice were subjected to surgical procedure, a thread was passed below both carotid arteries but the arteries were not occluded. After 15 min, the thread was removed and the animal was sutured back and allowed to recover for 24 h.
- Group II: Control (vehicle-pre-treated) group (*n* = 8). Mice were orally administered vehicle (simple syrup I.P. +Tween80 (5%), 10 mLkg^−1^) 1 h prior to being subjected to 15 min of global cerebral ischemia by bilateral carotid artery occlusion (BCAO) followed by reperfusion for 24 h.
- Group III: EGb761-treated group as positive control (*n* = 8). Mice were orally administered EGb761 at a dose of 80 mg kg^−1^, 1 h before subjecting them to global cerebral ischemia.
- Group IV: Drug extract-treated group (*n* = 8). Mice were orally administered methanol extract of MS (MS) at a dose of 100 mg kg^−1^, 1 h before subjecting them to global cerebral ischemia.
- Group V: Drug extract-treated group (*n* = 8). Mice were orally administered MS at a dose of 200 mg kg^−1^, 1 h before subjecting them to global cerebral ischemia.

Pilot experiments using infarct size as the end point were performed to choose the doses.

### 2.5. Bilateral Carotid Arteries Occlusion

Animals were subjected to BCAO under chloral hydrate anesthesia (400 mg kg^−1^ i.p.). A cotton thread was passed below each carotid artery. Pulling the ends of the thread with constant weight induced global cerebral ischemia. After 15 min of global cerebral ischemia, the weight on the thread was removed to allow reflow of blood through carotid arteries and the incision was sutured back in layers [32]. Temperature was maintained at 37°C throughout the surgical procedure.

### 2.6. Cerebral Infarct Size Assessment

After 24 h of reperfusion, animals were sacrificed by spinal dislocation and the brain was removed. The brain was kept overnight at −4°C. Frozen brain was sliced into uniform sections of *∼*1-mm thickness. The slices were incubated in 1% TTC at 37°C in 0.2 M tris buffer (pH 7.4) for 20 min [33]. TTC is converted to red formazone pigment by nicotinamide adenine dinucleotide (NAD) and dehydrogenase present in living cells. Hence viable cells were stained deep red. The infarcted cells loose these enzymes and, thus, remained unstained dull yellow. After the end of staining, color images of these slices were captured using a digital camera (Kodak 7230) and the software Adobe Photoshop 7.0. The size of infarct was calculated with the computerized image analyzer (MCID Elite). Percentage infarct volume was calculated using the following equation:

(1) $$\%\text{ Infract volume}\;\;=[(\frac{\text{Infract volume}}{\text{Total volume of }\;\text{slice}})\times 100].$$

### 2.7. Biochemical Analysis

#### 2.7.1. Estimation of TBARS

The brain was weighted, minced and suspended in a buffer containing 30 mM Tris-hydrochloric acid (HCl) and 2.5 mM calcium chloride (CaCl_2_ (pH 7.6)). The above mixture was homogenized, and the homogenate was centrifuged at 750 g to separate cellular debris. The supernatant was accurately divided into two parts. Both portions were centrifuged at 8200 g to obtain mitochondrial fraction. One was utilized for determination of TBARS [34] and the other one was employed for protein estimation [35].

#### 2.7.2. XO Activity and Superoxide Radical Generation in Xanthine-XO System

The brain was homogenized in ice-cold saline or phosphate buffer (50 mM, pH 7.8). The homogenate in phosphate buffer containing ethylene diaminetetraacetic acid (EDTA) 1 mM, DTT 10 mM and PMSF 1 mM, was centrifuged at 25 000 g for 30 min at 4°C [36]. The supernatant was used for determination of XO activity and O^•−^_2_ production. The 25 000 g supernatant from the XO homogenizing buffer was used for determinations of the enzymatic activities of XO, total (XO + XD) and %XO of the total [37]. Another aliquot of the supernatant was used for the O^•−^_2_ production [38], expressed as pmol min^−1^ mg^−1^ of protein, based on an extinction coefficient Δ*ε*550 = 21 × 103 M^−1^ cm^−1^ [39].

#### 2.7.3. Superoxide Dismutase

SOD activity was measured by the method of Kakkar et al. [40]. The mice brains were homogenized in ice-cold sodium pyrophosphate buffer (pH 8.3) in a ratio of 50 mg mL^−1^, and 200 *μ*L of this homogenate was used for the assay. The inhibition by SOD of reduction of NBT to blue-colored chromogen in the presence of PMS and NADH was measured at 560 nm. One unit of enzyme activity was defined as enzyme concentration required to inhibit the absorbance at 560 nm of chromogen production by 50% in 1 min under assay conditions, and expressed as specific activity in unit of SOD min^−1^ mg^−1^ of protein.

#### 2.7.4. Reduced Glutathione

GSH in the brain was determined by the method of Jollow et al. [41]. A total of 1 mL of PMS (10% w/v) was precipitated with 1 mL of sulfosalicylic acid (4%). The samples were kept at 4°C for at least 1 h and, then, subjected to centrifugation at 1200 g for 15 min at 4°C. The assay mixture contained 0.1 mL of PMS (10%, w/v), 2.7 mL of phosphate buffer (0.1 M, pH 7.4) and 0.2 mL DTNB (40 mg/10 mL phosphate buffer, 0.1 M, pH 7.4) in a total volume of 3 mL. The yellow color that developed was read immediately at 412 nm. The enzyme activity was calculated as nM DTNB oxidized/min per milligram of protein.

#### 2.7.5. T-SH Groups Assay

This assay was carried out following the method of Sedlack and Lindsay [42]. Briefly, a 200-mg sample of brain tissue was homogenized in 8 mL of 0.02 M EDTA, and 500 *μ*L of this was used for the assay. Measurement of T-SH was done by analyzing the reaction of SH groups in tissue with DTNB and levels are expressed as mmol SH mg^−1^ of proteins.

### 2.8. Behavioral Testing

#### 2.8.1. Short-Term Memory Evaluation Using Elevated Plus Maze

Plus maze consists of two enclosed (16 × 5 × 12 cm) and two open (16 × 5 cm) arms, connected by a central platform (5 × 5 cm) [43]. It was elevated to a height of 25 cm above the floor. All the animals were given a single trial on the plus maze. Each mouse was placed individually at the end of the open arm facing away from central platform of the maze. The time taken by the mouse to enter from the open arm with all the four legs into the enclosed arm was taken as transfer latency time (TLT) [44, 45].

#### 2.8.2. Inclined Beam Walking Test

To evaluate fore and hind limb motor coordination, inclined beam walking test was employed [46]. Inclined beam walking test was performed before ischemia and 24 h after reperfusion.

### 2.9. Antioxidant Activity of MS In Vitro

#### 2.9.1. Superoxide Anion-Scavenging Activity

Superoxide anion-scavenging activity of the extracts was validated based on the method described by Liu et al. [47] with slight modification [48]. The superoxide radical is generated in PMS-NADH systems by oxidation of NADH and assayed by the reduction of NBT. In this experiment, the superoxide radical was generated in 3 mL of Tris-HCl buffer (16 mM, pH 8.0) containing 1 mL of NBT (50 *μ*M), 1 mL NADH (78 *μ*M) and sample solution of extract (from 25 to 75 *μ*g mL^−1^) in water were mixed. The reaction was started by adding 1 mL of 10 M PMS to the mixture. The reaction mixture was incubated at 25°C for 5 min, and the absorbance was recorded at 560 nm against blank samples. l-Ascorbic acid was used as a control. The percentage inhibition of O^•−^_2_ generation was calculated using the following formula:

(2) $$\text{Inhibition}\;\;(\%)=\;\{\frac{\left(A_{0}-A_{1}\right)}{A_{0}}\}\times 100,$$

where *A*_0_ was the absorbance of the control (l-ascorbic acid), and *A*_1_ was the absorbance of drug extract and standards.

#### 2.9.2. Free Radical-Scavenging Activity

The free radical-scavenging activity of MS was measured by DPPH radical using the method of Shimada et al. [49]. Briefly, 0.1 mM solution of DPPH in ethanol was prepared and 1 mL of this solution was added to 3 mL of sample solution of extract in water at different concentrations (25–75 *μ*g mL^−1^). The absorbance was measured at 517 nm. The percent DPPH radical-scavenging effect was calculated using the following formula:

(3) $$\begin{array}{cc} & \text{DPPH}\;\text{ radical scavenging effect }(\%)\\ & \;=100-[\{\frac{\left(A_{0}-A_{1}\right)}{A_{0}}\}\times 100], \end{array}$$

where *A*_0_ was the absorbance of the control reaction and *A*_1_ was the absorbance in the presence of the standard sample or extract.

### 2.10. Statistical Analysis

All the data are presented as mean ± SEM. The data of infarct size and biochemical parameters were statistically analyzed using one-way analysis of variance (ANOVA) followed by *post hoc* Tukey's multiple range test. The data of percentage change in TLT was statistically analyzed using repeated-measure ANOVA followed by *post hoc* Tukey's multiple range test. Inclined beam walking test was statistically analyzed using one-way ANOVA followed by *post hoc* Kruskal-Wallis test. A *P*-value of <.05 was considered statistically significant.

## 3. Results

### 3.1. Neuroprotective Effects of MS

**Figure 1:**
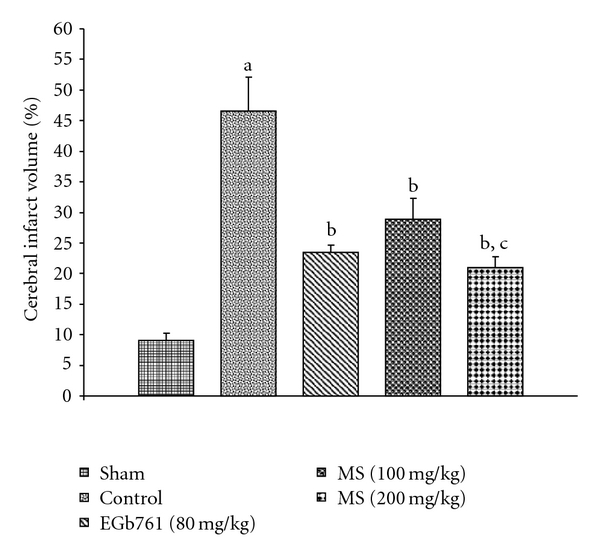
Effect of methanol extract of MS on brain infarction in mice subject to global cerebral ischemia followed by reperfusion. Each column represents the mean ± SEM, *n* = 8. ^a^ *P* < .05 versus Sham; ^b^ *P* < .01 versus control (vehicle pre-treated); ^c^ *P* < .05 versus 100 mg kg^−1^, p.o. of the extract.

Global cerebral ischemia of 15 min followed by reperfusion for 24 h produced significant increase in cerebral infarct size. Administration of MS (100 or 200 mg kg^−1^) 1 h before ischemia markedly attenuated I/R-induced increase in cerebral infarct volume by 37.9 and 54.8%, respectively (*P* < .01; Figure 1). Administration of EGb761 (80 mg kg^−1^) similarly reduced the infarct volume by 49.6% (*P* < .01; Figure 1). This suggested that the administration of MS exhibited protective effect against I/R induced cerebral injury, when compared to control.

### 3.2. Antioxidant Effects of MS In Vivo

**Figure 2:**
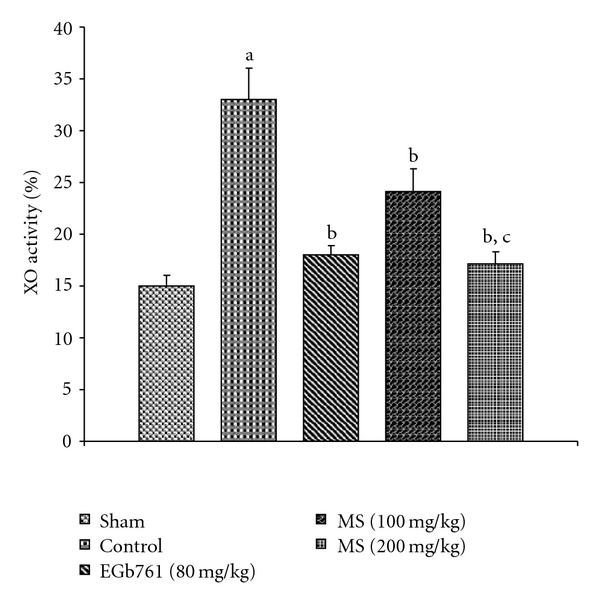
Effect of methanol extract of MS on % XO activity in mice subject to global cerebral ischemia followed by reperfusion. Values expressed as mean ± SEM, *n* = 8. ^a^ *P* < .05 versus Sham; ^b^ *P* < .05 versus control (vehicle pre-treated); ^c^ *P* < .05 versus 100 mg kg^−1^, p.o. of the extract. % XO = [XO/(XO + XD)] × 100.

**Figure 3:**
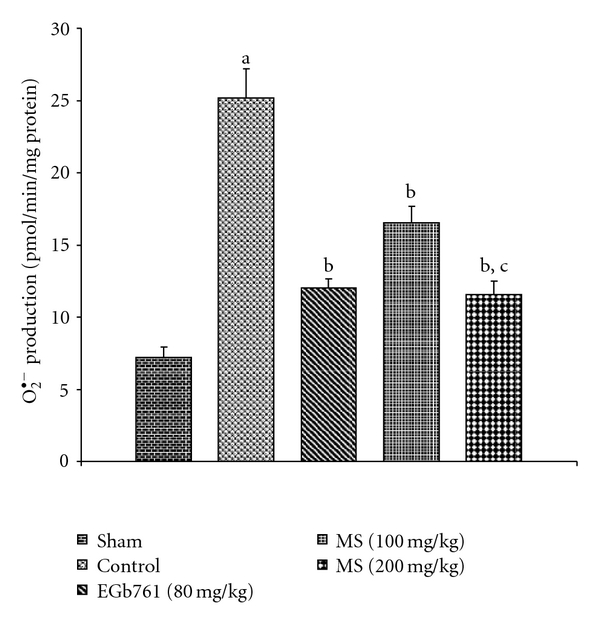
Effect of methanol extract of MS on superoxide anion (O^•−^_2_) production in mice subject to global cerebral ischemia followed by reperfusion. Values expressed as mean ± SEM, *n* = 8. ^a^ *P* < .05 versus Sham; ^b^ *P* < .05 versus control (vehicle pre-treated); ^c^ *P* < .05 versus 100 mg kg^−1^, p.o. of the extract.

**Figure 4:**
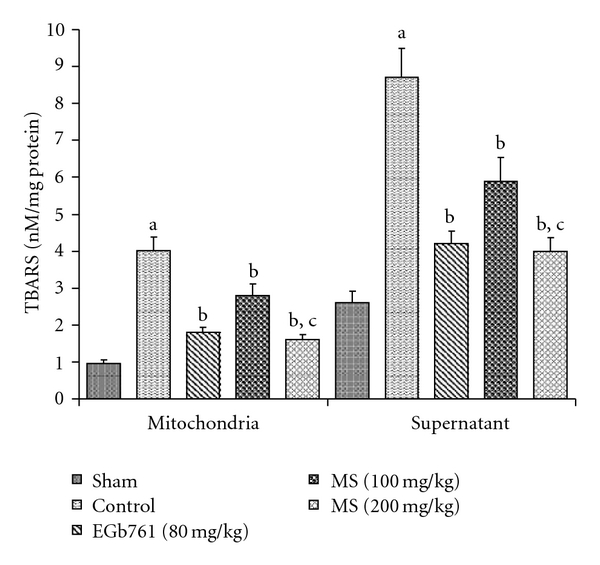
Effect of methanol extract of MS on mitochondrial and supernatant TBARS formation in mice subject to global cerebral ischemia followed by reperfusion. Each column represents the mean ± SEM, *n* = 8. ^a^ *P* < .05 versus Sham; ^b^ *P* < .05 versus control (vehicle pre-treated); ^c^ *P* < .05 versus 100 mg kg^−1^, p.o. of the extract.

Figure 2 depicts that I/R insult (control group) resulted in >2-fold increase in % XO of total (XO + XD) activity in mouse brain compared with the sham group. This increase was effectively diminished by 48.2% of control group after administration of MS (200 mg kg^−1^). The production of O^•−^_2_ Paralleled the increase in % XO activity in mouse ischemic brain where *∼*3-fold increase in O^•−^_2_ production was noted compared to the sham level (Figure 3). MS significantly hampered this free radical burst to about 54% of the elevated levels of the control group.  Figure 4 shows that TBARS concentration in brain mitochondria and supernatant fractions was significantly elevated in mouse ischemic brain due to I/R insult. Pre-treatment with MS significantly decreased the elevated TBARS concentration in brain mitochondria and supernatant fractions as compared to control group. Likewise, EGb761 (80 mg kg^−1^) significantly suppressed I/R-induced increase in XO, O^•−^_2_ production and TBARS concentration to a similar extent.

All biochemical parameters (GSH, SOD and T-SH) showed a significant decrease in the control group versus the respective sham group. Pre-treatment with MS markedly reversed the alterations in biochemical parameters brought about by I/R. The values were almost restored to near normal levels with no significant differences versus the sham group. GSH, SOD and T-SH were significantly elevated (rather than showing depletion) in the MS-treated animals subjected to BCAO and reperfusion injury as compared to the control group (Table 1). Pre-treatment with EGb761 (80 mg kg^−1^) significantly suppressed oxidative stress to a similar extent.

### 3.3. Antioxidant Activity of MS In Vivo

**Figure 5:**
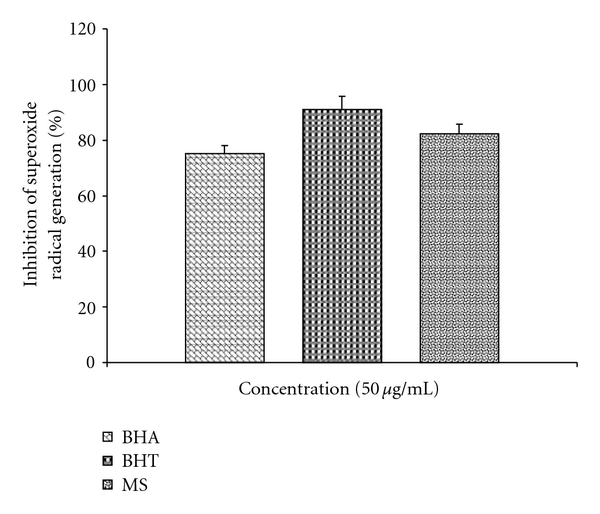
Comparison of superoxide anion radical-scavenging activity of MS, BHA and BHT by the PMS-NADH-NBT method at the concentration (50 *μ*g mL^−1^). BHA = butylated hydroxyanisole; BHT = butylated hydroxytoluene. Values expressed as mean ± SEM.

**Figure 6:**
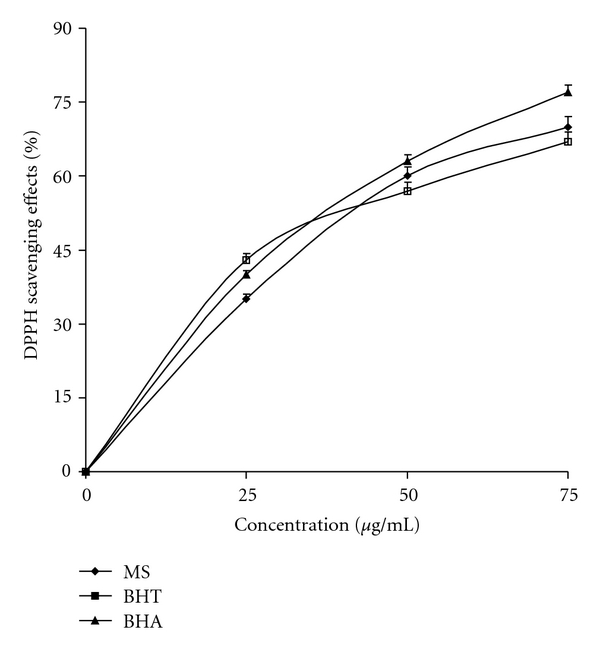
Free radical-scavenging activity of different concentrations of MS, BHA and BHT by DPPH radicals. BHA = butylated hydroxyanisole; BHT = butylated hydroxytoluene. Values expressed as mean ± SEM.

Direct antioxidant activity by MS is shown in Figures 5 and 6. At concentrations of 25–75 *μ*g mL^−1^, MS exhibited concentration-dependent scavenging activities against O^•−^_2_ generated in PMS-NADH systems. MS showed significant superoxide radical-scavenging activity, exhibited higher superoxide radical-scavenging activity than BHA, but lower than BHT (*P* < .05) at the same concentration of 50 *μ*g mL^−1^, where BHA and BHT were used as standard radical scavengers. In another assay, the free radical-scavenging activity of MS was measured in terms of hydrogen-donating or radical-scavenging ability against a stable radical DPPH. The scavenging effect of MS and standards on the DPPH radical decreased in the order of BHA > MS > BHT and were 77, 70 and 67% at the concentration of 75 *μ*g mL^−1^, respectively. These results indicated that MS have a significant effect on scavenging free radicals.

### 3.4. Behavioral Observations

**Figure 7:**
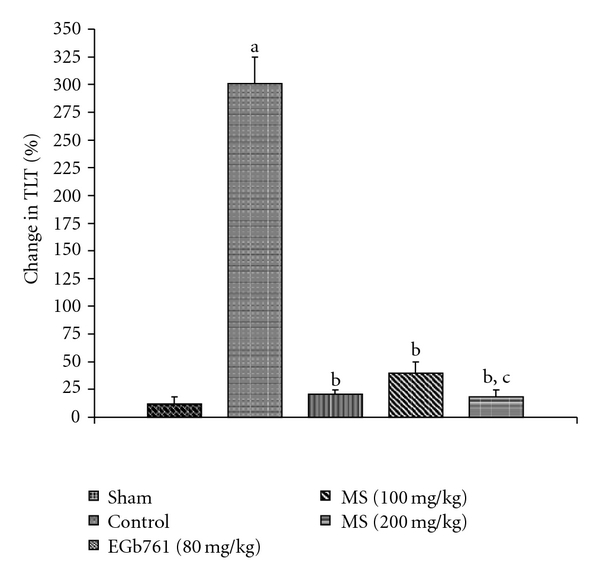
Effect of methanol extract of MS on impairment of short-term memory (% change in TLT) in mice subject to global cerebral ischemia followed by reperfusion. Values expressed as mean ± SEM, *n* = 8. ^a^ *P* < .05 versus Sham; ^b^ *P* < .05 versus control (vehicle pre-treated); ^c^ *P* < .05 versus 100 mg kg^−1^, p.o. of the extract.

**Figure 8:**
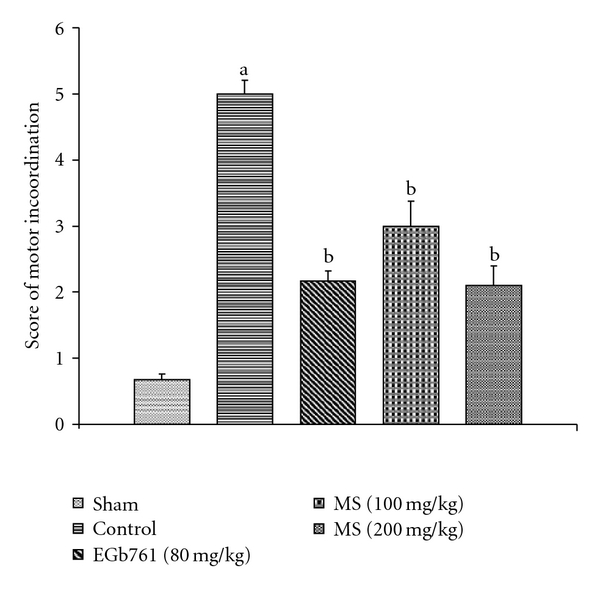
Effect of methanol extract of MS on impairment of motor performance (score of motor incoordination) in mice subject to global cerebral ischemia followed by reperfusion. Values expressed as mean ± SEM, *n* = 8. ^a^ *P* < .05 versus Sham; ^b^ *P* < .05 versus control (vehicle pre-treated).

The training trials performed on day 2 and day 3 significantly decreased the TLT as compared with TLT on day 1 using elevated plus maze. Pre-treatment with MS significantly prevented I/R-induced increase in the percentage change in TLT (Figure 7). Global cerebral ischemia also produced a marked impairment of motor performance. MS treatment before I/R significantly prevented I/R-induced impairment of motor performance (Figure 8). Similarly, EGb761 pre-treatment markedly attenuated behavioral deficits.

## 4. Discussion

In the present study, the antioxidant and neuroprotective potential of MS was studied against BCAO-induced oxidative stress in mice. Experimental models of stroke have been developed in animals in an attempt to mimic the events of human cerebral ischemia. It is well documented that transient global cerebral ischemia results in neurological abnormality. Therefore, global cerebral ischemia of short duration followed by reperfusion has been employed in the present study.

EGb761, a patented extract from the leaves of the *Ginkgo biloba*, is a scientifically proven natural product widely used to treat cerebral ischemic disorder and neurological disorder [13, 50]. EGb761 was used as a reference to examine the neuroprotective abilities of MS in mice from I/R injury. The present investigations revealed that MS exhibited neuroprotective activity similar to that shown by EGb761. Pre-treatment with MS markedly reduced the infarct size induced by BCAO and produced significant protection against neuronal damage (Figure 1), in harmony with other studies [51, 52].

TBARS, GSH, SOD and T-SH were estimated as an index to assess the severity of oxidative damage in the brain tissue, and also the effect of MS on the reversal of the damage produced by BCAO. All these parameters were markedly reversed and restored to near normal levels in the groups pre-treated with MS.

Free radicals are well investigated in the development of I/R-induced cerebral injury [53, 54]. ROS produces malondialdehyde (MDA), an end product of lipid peroxidation (LPO). MDA reacts with TBA and is, thus, estimated as TBARS [55]. Therefore, MDA was estimated using TBARS assay to estimate extent of ROS. During ischemia, XD undergoes irreversible proteolytic conversion to XO, producing O^•−^_2_ and H_2_O_2_ in the presence of oxygen [53]. O^•−^_2_ does not directly induce LPO but can react with NO^•^ to form cytotoxic ONOO^−^ [56]. In addition, it is well documented that XO is an important prerequisite factor in the process of O^•−^_2_ generation in acute post-ischemic reperfusion injury [5, 57]. Such finding is in harmony with our results where a significant rise in % XO was found in mouse brain after I/R insult. The production of O^•−^_2_ paralleled the rise in % XO activity where a significant increase in O^•−^_2_ generation resulted in untreated control group compared to the sham group. In the current study, the elevated % XO activity of the untreated control group was effectively counteracted by administration of MS. Moreover, it also significantly hampered O^•−^_2_ burst to about half the value of the untreated control group. One proposed mechanism underlying this protective effect of MS, in the model used in the present investigations, could be through its antioxidant properties, restoring endogenous antioxidant levels and, therefore, detoxifying free radicals.

The overproduction of free radicals can be detoxified by endogenous antioxidants causing their cellular stores to be depleted [58]. Glutathione is considered a central component in the antioxidant defenses of cells. It acts both to directly detoxify ROS and as a substrate for various peroxidases [59]. Moreover, it is well evidenced that SOD activity in serum is reduced in stroke patients, and replacement of antioxidant activity could be beneficial in the acute treatment of cerebral ischemia [60]. Similarly, fall in T-SH reflects the consumption of tissue thiols. As such, fall in GSH during cerebral reperfusion injury is well reported [61]. Sulfhydryl compounds are considered important endogenous antioxidants. They have role in maintenance of cellular proteins and lipids in their functional states [62]. Pre-treatment with MS significantly elevated GSH, SOD and prevented fall in T-SH in control groups.

Hippocampal neurons, which are involved in the regulation of short-term memory, are highly susceptible to I/R-induced injury [63]. Therefore, elevated plus maze has been employed in present study to evaluate impairment of short-term memory as a result of cerebral I/R. Cerebral ischemia is documented to impair sensory motor ability [64] and thus inclined beam walking test has been used in the present study to investigate the effect of cerebral I/R on motor performance.

In conclusion, the present findings suggest a potential role of MS in cerebral stroke, and the findings are important in view of the fact that stroke is at present the second leading cause of death worldwide [65]. The mechanisms by which MS normalized the cerebral damage and stress, and prevented impairment of short-term memory and motor incoordination, is probably the antioxidant property of the drug which was evaluated *in vitro*, to direct scavenged free radical generated against a stable radical DPPH and O^•−^_2_ radical generated in PMS-NADH systems, and its inhibitory effects on XD/XO conversion and resultant O^•−^_2_ production. Further studies are warranted to pursue the interesting lead emerging from the present results to exploit the full therapeutic potential of MS in cerebrovascular diseases.

## Funding

Financial support from the L.L.R. Educational Trust, Solan, Himachal Pradesh, India, which runs the L.R. Institute of Pharmacy, is gratefully acknowledged.

## Figures and Tables

**Table 1:** Effect of MS methanol extract on biochemical parameters during cerebral post-ischemic reperfusion (15-min BCAO and 24-h reperfusion).

Group	SOD (U mg^−1^ protein)	T-SH (×10^−5^ M mg^−1^ protein)	GSH (nM mg^−1^ protein)
Sham	102.01 ± 17.91	3.59 ± 0.27	5.51 ± 0.34
Control	65.90 ± 14.39**	2.34 ± 0.26*	2.20 ± 0.92**
EGb761 (80 mg kg^−1^)	97.21 ± 10.52**	3.42 ± 0.20***	5.54 ± 0.51**
MS (100 mg kg^−1^)	84.45 ± 9.61**	3.29 ± 0.35**	3.75 ± 0.61***
MS (200 mg kg^−1^)	97.81 ± 12.71*	3.47 ± 0.25***	5.57 ± 0.46**

All data are expressed as mean ± SEM, *n* = 8. Sham and treatment groups are compared with control (vehicle pre-treated). *P< .001, **P< .01, ***P< .05. Statistical analysis was done by one-way ANOVA followed by Tukey's Test.
